# Attribution of sensory prediction error to perception of muscle fatigue

**DOI:** 10.1038/s41598-022-20765-9

**Published:** 2022-10-06

**Authors:** Sho Ito, Toshitaka Kimura, Hiroaki Gomi

**Affiliations:** 1 NTT Communication Science Laboratories, Nippon Telegraph and Telephone Corporation, Atsugi, Kanagawa Japan; 2 School of Engineering, Tokyo Institute of Technology, Yokohama, Kanagawa Japan

**Keywords:** Perception, Consciousness

## Abstract

Sensory prediction-error is vital to discriminating whether sensory inputs are caused externally or are the consequence of self-action, thereby contributing to a stable perception of the external world and building sense of agency. However, it remains unexplored whether prediction error of self-action is also used to estimate the internal body condition. To address this point, we examined whether prediction error affects the perceived intensity of muscle fatigue. Participants evaluated fatigue while maintaining repetitive finger movements. To provide prediction error, we inserted a temporal delay into online visual feedback of self-movements. The results show that the subjective rating of muscle fatigue significantly increased under the delayed visual feedback, suggesting that prediction error enhances the perception of muscle fatigue. Furthermore, we introduced visual feedback that preceded actual finger movements to test whether the temporal direction of the mismatch is crucial in estimating muscle fatigue. We found that perceived fatigue was significantly weaker with preceding visual feedback compared to normal feedback, showing that the perception of muscle fatigue is affected by the signed prediction-error. Our findings support the idea that the brain flexibly attributes prediction errors to a self-origin with keeping sense of agency, or external origin by considering contexts and error characteristics.

## Introduction

Even in an ever-changing environment, we can correctly perceive the external world and accurately move our bodies by extracting the necessary information from noisy sensory inputs. As an underlying mechanism of this essential function, earlier pioneering works^1,2^ demonstrated that the brain predicts the sensory consequences of action using an efference copy of the motor command. It is believed that the brain uses the error between this prediction and the actual sensory input for online motor correction^3^ and motor learning^4^.

In addition to the pivotal role in motor control, growing evidence suggests that the prediction error also crucially contributes to detecting and estimating physical influences from the external world. For example, numerous studies have shown that we perceive sensory inputs accompanied by self-movement as less intense than stimuli generated by others^5,6^. This ‘sensory attenuation’ of self-produced stimuli is thought to be a mechanism to direct attention to external events by suppressing the sensory consequence of one’s own action^5,7^. Adding a spatial or temporal mismatch between the action and its feedback eliminates this sensory attenuation^8–10^, demonstrating that the brain utilizes the sensory prediction error to discriminate external events from self-caused sensory inputs. Similarly, a spatiotemporal mismatch between the expected and actual result of an action reduces the resulting sense of agency^11,12^, suggesting that prediction errors can lead sensory events to be attributed to other agents. Furthermore, sensory prediction error modulates the estimation of the physical properties of external objects or environments. Altering the visual feedback of voluntary movements changes the perceived weight^13–15^ and dynamics^16^ of held objects, and the mechanical impedance of environments^17,18^. These perceptual modulations are interpreted as resulting from attributing the prediction error to changes in the properties of external objects.

In contrast to the prevailing view, which postulates a tight association between prediction errors and extrinsic factors, we offer the more generalized idea that prediction errors can often be caused by changes in one’s own condition (intrinsic factors) as suggested by a recent theory regarding interoception^19–21^. Human motor function is dynamically affected by various factors related to self-condition, including body growth, aging^22^, injury^23^, muscle contraction history^24^, and fatigue^25,26^. Until the internal model is updated, these intrinsic sensorimotor changes cause executed movements to differ from predicted actions, resulting in error signals. Accordingly, it is possible that these prediction errors calculated using exteroceptive signals can be used to estimate changes in one’s bodily condition, rather than being solely for detecting external change.

In the present study, we particularly focus on muscle fatigue, which induces an acute decline in motor function. Muscle fatigue is defined as an exercise-induced decrease in the capability to exert force^25^, and it can significantly affect the performance of voluntary action^27^. Our working hypothesis is that the brain will ascribe prediction error computed with exteroceptive information to reduced motor output due to muscle fatigue (i.e., performance fatigability^28^), providing the given context indicates fatigue is likely to occur. Consequently, during a motor task, the perceived intensity of muscle fatigue (i.e., perception of fatigue^28^) is hypothesised to be affected by prediction error. To provide the prediction error, we inserted a delay into online visual feedback of a finger movement. To further investigate how prediction error is processed, we tested the effect of a temporal shift in the opposite direction, namely, negative lag of visual feedback. Exploiting a property of repetitive movement and a prediction algorithm for human cyclic movements^29^, we generated artificial feedback which preceded the ongoing finger movement.

Through two experiments, we found that the intensity of perceived fatigue was enhanced by delayed visual feedback, but was reduced by preceding visual feedback. These results suggest that, dependent on context, the brain can attribute the sensory prediction error calculated from exteroceptive signals to internal factors, including changes in bodily condition.

## Methods

In total, 28 naïve right-handed participants (21 females, 7 males; age range 20–42, average 30 ± 6.0) participated in either of the two experiments. Fourteen of them participated in the first experiment, and the other 14 participants participated in the second experiment. All participants gave written informed consent. All of the experimental protocols were approved by the ethics committee of NTT Communication Science Laboratories and were performed in accordance with the Declaration of Helsinki. The sample size of Experiment 1 was determined based on previous studies^8,15^. After Experiment 1, since we calculated the minimum sample size as being 10, using an effect size from the main results (the difference between fatigue choice rate at 50 ms delay and chance level, *d* = 1.00, with parameters of *α* = 0.05 and *β* = 0.8), we held the sample size the same in Experiment 2.

**Figure 1:**
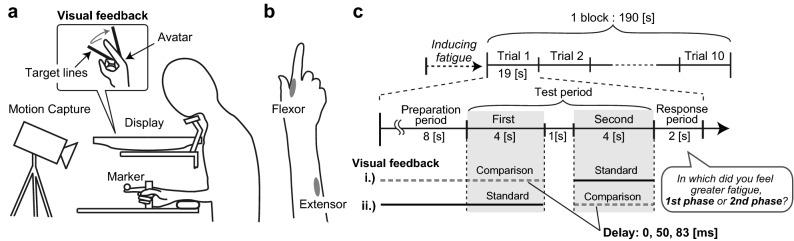
Experimental procedure. (**a**) Experimental setup. (**b**) Illustration of EMG electrode placement. (**c**) Procedure of Experiment 1. Measurements started soon after participants felt sufficient muscle fatigue induced by rapid voluntary finger movement. They repeated cyclic finger flexion and extension at 2.14 Hz without a break during an experimental block consisting of 10 successive trials. In the response period at the end of each trial, participants were asked to indicate during which of the two phases of the last test period they felt greater muscle fatigue. Metronome beeps to pace the finger movements were provided in the preparation period of all trials. In half of the trials, the beeps were also provided during the test period (sound-on trial), while they were not provided in the other half (sound-off trial). Visual feedback of the finger movement was delayed (0, 50, 83 ms) in phase of the test period. Visual feedback was not presented between the first and second phases of the test period, nor during the response period. In a later session, participants also judged the visual delay with the identical procedure, where they were asked to select in which phase of the test period they perceived a larger delay of the visual feedback.

Participants sat on a chair with their right arm resting on the horizontal armrest and held a vertical handle (Fig. 1a). Their hand was occluded from their view by a PC monitor (LCD-MF221X, I-O data device Inc., Ishikawa, Japan; resolution: 1920 × 1080 pixels; refresh rate: 60 Hz) horizontally placed over the workspace. Their head position was supported by a chinrest to keep the distance between the eye and the display at approximately 30 cm. Instead of direct vision of the hand, a hand avatar (picture of right hand with index finger extended) was displayed on the monitor using MATLAB (Mathworks Inc, Natick, MA) and Cogent graphics toolbox (developed by John Romaya at the LON at the Wellcome Department of Imaging Neuroscience). The position of the avatar was visually aligned to the actual hand location. Participants’ finger position was detected with a reflective marker placed on the fingertip at a rate of 500 Hz using a motion capture system (ProReflex, Qualisys Co., Sweden). This data was resampled at 60 Hz and used to display the avatar’s index finger so that its flexion angle matched synchronously with the actual finger movement. Latency from the finger movement detection to showing the avatar, measured by using a photodetector, was 57 ± 1.1 ms. The task was to cyclically continue flexion and extension of their index finger with the distal and proximal interphalangeal joints extended between two target lines drawn from the rotation center of the metacarpophalangeal joint. The angle between the target lines was adjusted for each participant (38°–55°) to provide an appropriate fatigue level with the following procedure. Prior to the experiment, we asked participants to perform the task with the same procedure as during the actual experiment (i.e., after inducing sufficient muscle fatigue) and asked the trend of fatigue they perceived (i.e., increasing, decreasing or constant). Depending on their answer, we repeatedly modified the angle between the lines (e.g., we decreased the angle if fatigue increased too rapidly to continue the task) until each participant felt moderate fatigue throughout the block. To pace the movement, we provided an auditory metronome at a frequency of 2.14 Hz. Participants were required to produce finger flexion and extension between every set of two tones. Though the metronome tones were removed during part of the trials to examine their effect on fatigue perception, participants were instructed to repeat the movement at the same pace.

Electromyogram (EMG) signals were recorded from the flexor (First dorsal interosseous) and the extensor (Extensor digitorum) muscles of the right index finger with surface electrodes (Fig. 1b). The electrode locations were determined by palpation of the muscle belly during flexion and extension of the index finger. These signals were amplified (EMG-021, Harada Electronic Industry Ltd., Hokkaido, Japan), filtered (10–1000 Hz), and then recorded at a sampling rate of 500 Hz. For later analysis, the signals were rectified after band-pass filtered (20–240 Hz, 4th order Butterworth filter). We chose this frequency band considering that the EMG power rapidly decreases above 100 Hz and becomes substantially small (< − 20 dB) around 250 Hz^30–32^, and that this band includes adequate signal to calculate the median frequency (typically less than ~ 120 Hz^33^) for monitoring the level of muscle fatigue^34^ (see “Data analysis” section). We excluded one participant in Experiment 1 and two participants in Experiment 2 from all EMG analyses because signal quality was poor due to electrode problems.

Before the experiments, to make the participant experience evident muscle fatigue (i.e., performance fatigability), we had them repeat rapid flexion and extension of the index finger until the finger movement clearly slowed down (typically, it took several tens of seconds). Then, we asked them to freely describe a sensation that they felt around the index finger. The answers varied depending on participants, such as “sluggish sensation”, “heaviness in the finger”, “feeling the finger stiffer”, and so on. We defined the sensation each participant reported as a measure of fatigue perception in the experiment. Participants were instructed to focus attention on that sensation when evaluating their perception of fatigue throughout the following experiment.

### Experiment 1

Before each experimental block, participants were instructed to repeatedly flex and extend their right index finger as rapidly as possible to induce muscle fatigue. After the participants verbally reported that they started to feel clear fatigue, they were told to pace their finger movement using the metronome sound. Soon after that, the experimental block started. A block (190 s) consisted of 10 consecutive trials (Fig. 1c), and participants were required to continue the instructed finger movement without any breaks in each block. Each trial (19 s) consisted of a preparation period (8 s), test period (9 s), and response period (2 s).

During the preparation period, a metronome was presented to enable participants to pace their finger movements. Participants were asked to compare the fatigue perceived during the first phase of the test period and that perceived during the second phase of the test period. In the subsequent response period, participants were required to choose on which phase they perceived greater fatigue in a two-alternative forced-choice manner, by pressing one of two keys with their left hand. The first and second phases of the test period were indicated by a change in the colors of the target lines (red or blue, order of the colors was counterbalanced within participants). In half of all trials (randomly ordered), the metronome was not presented in the test period (sound-off trials), while it was presented in the other half (sound-on trials). In either phase of the test periods, a delay was imposed on the movement of the finger avatar (comparison stimuli, 0, 50, and 83 ms), while in the other phase, it synchronously moved with the actual finger (standard stimuli). The visual feedback was not presented in the interval (1 s) between the first and second phases of the test period to avoid an unnatural jump of the visual feedback due to adding or removing the delay. Note that in trials where the visual delay was assigned to the first phase, the delay was implemented from the beginning of the preparation period. In pilot experiments, we observed that participants tended to increase the amplitude of the finger movement due to the delayed visual feedback. Possibly, this tendency resulted from overshooting the target position due to the delay in feedback of hand state^35^. In order to rule out the possibility that a change in the movement amplitude affected the perception of fatigue, the angle between target lines was slightly reduced when the visual delay was imposed (− 3.6% for 50 ms delay, and − 6% with 83 ms delay). We confirmed that all participants were not aware of that change in target lines by asking them verbally after the experiments.

Every experimental block contained five sound-on trials and five sound-off trials. Both of them consisted of five different types of trials regarding the presentation of comparison stimulus (50 ms delay on the first phase, 50 ms delay on the second phase, 83 ms delay on the first phase, 83 ms delay on the second phase, and 0 ms delay on either phase). The order of these ten trials was randomized. Thirteen participants performed 15 experimental blocks, and one participant did 12 blocks.

After the main experiment (fatigue evaluation session), we also measured the ability to detect the delay in visual feedback (delay detection session), using the same procedure as that of the main experiment. In this session, we asked participants to choose which half of the test period they perceived to contain more visual feedback delay. All participants performed 12 experimental blocks in this session.

### Experiment 2

**Figure 2:**
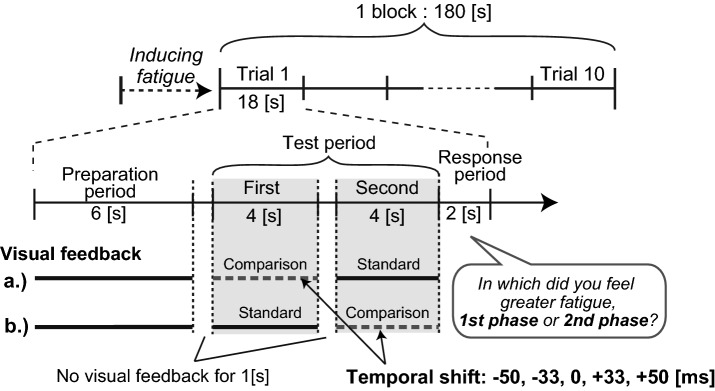
Procedure of Experiment 2. As in Experiment 1, participants maintained cyclic finger movement (2.14 Hz) throughout an experimental block consisting of 10 seamless trials (18 s), after feeling sufficient muscle fatigue. On every trial, they were asked to evaluate the intensity of perceived fatigue by comparing the first and the second phase of the test period. In either phase of the test period, delayed or preceding visual feedback (− 50, − 33, 0, + 33, + 50 ms) was provided (comparison), while visual feedback was displayed as it was in the other phase (standard). Visual feedback was not presented between the preparation and test periods, between the first and second phases of the test period, or during the response period. Metronome beeps for pacing were provided in the preparation period and the response period, but not in the test period.

In the second experiment, we also tested the effect of negative lag (or preceding) of visual feedback on fatigue perception to clarify if the prediction error in the opposite direction is also reflected in the attribution process. We provided visual feedback which preceded ongoing finger movement using a prediction technique for human cyclic motion (see Supplementary Information for details). The experimental sequence was almost identical to Experiment 1 except for several minor changes (Fig. 2). In Experiment 2, the length of the experimental block (180 s) was shorter than Experiment 1 due to a shortened preparation period in each trial. To control eye position, we displayed a fixation cross 9.5 cm to the left of the finger avatar location. Throughout an experimental block, the participants were instructed to see the fixation cross. In either half of the test periods, positive and negative lag was imposed on the movement of the finger avatar (comparison stimuli, − 50, − 33, 0, + 33, and + 50 ms, randomly ordered), while in the other half, the avatar movement was displayed without any additional lags (standard stimuli). As in Experiment 1, the participants were asked to select the half of the test period in which they felt greater muscle fatigue. To control the amplitude of finger movements, we changed the angle between target lines when the comparison stimuli were displayed (− 2.4% for 50 ms delay, and − 1.6% with 33 ms delay). In a part of trials with the negative lag, prediction accuracy of the preceding feedback was not desirable because of the large variability of finger movements. Thus, we excluded the trials where prediction performance became lower than a threshold (state variable ${\omega }_{t}$ < 0.05, see Supplementary Information). In total, 7.0% of negative lag trials were excluded from the analysis. In every experimental block, we presented each comparison stimuli (− 50, − 33, 0, + 33, and + 50 ms) once for each phase of the test period in a pseudo-randomized order. Each participant performed 15 experimental blocks.

As in Experiment 1, in a separate session, we also examined how accurately the positive and negative delay of visual feedback is detected using a similar procedure as employed in the main experiment. Participants were asked to select in which phase of the test period they perceived larger asynchrony of visual feedback. Seven levels of temporal shift of visual feedback (− 50, − 33, − 17, 0, + 17, + 33, + 50 ms) were provided as comparison stimuli. All the participants performed ten experimental blocks consisting of 14 trials. In this session, we did not apply the fatigue-induction exercise before each experimental block.

### Data analysis

We calculated the choice probabilities of the comparison stimuli for both the fatigue evaluation session and the delay detection session. We merged responses to compare stimuli that were presented in the first and second phases of the test period to remove any bias due to presentation order, including a potential temporal trend within the trial. Bonferroni corrected *t*-tests were applied to compare these values with chance level. We conducted two-way repeated-measure ANOVA with the factor of visual delay and task (fatigue vs delay) to compare the choice probabilities between the sessions. We also examined the effect of the pacing sound on the fatigue evaluation session by conducting two-way repeated-measure ANOVA with the factor of visual delay and sound (sound-on vs sound-off).

To estimate the sensitivities of perceived fatigue and delay discrimination to visual delay in Experiment 1, we calculated the slope of each response. For both types of response, probabilities of choosing comparison stimuli against visual delay were fitted to sigmoid curves by probit regression. To consider both population-level trend and subject-specific variability in the response, we applied the Generalized Linear Mixed Model (GLMM) to the fitting^36^. Here, the response of participant $i$ is expressed as

1 $$\begin{array}{c}{P}_{i}\left(x\right)=\Phi \left(\alpha +{\alpha }_{i}+\left(\beta +{\beta }_{i}\right)x\right),\end{array}$$

where ${P}_{i}$ is the probability of the response, *x* is the amount of visual delay, $\Phi$ is the cumulative distribution function of the standard normal distribution, α and β are fixed-effect parameters of bias and slope of curves, ${\alpha }_{i}$ and ${\beta }_{i}$ are random-effects parameters determining participant-dependent bias and slope of curves, respectively. The fitting was performed using MATLAB function ‘fitglme’ with a parameter ‘Laplace’ to select the estimation method. After fitting data, we tested if the participant-level slope parameters $(\beta +{\beta }_{i})$ were correlated between perceived fatigue and visual delay detection.

Further, we used the GLMM framework to examine if the response pattern of the fatigue evaluation task was better explained by the response patterns during the visual delay discrimination task. In the model described in Eq. (1), we fitted the response probability in the fatigue evaluation session using the amount of visual delay as a prediction variable (Model 1). Similarly, we also fit the same data instead using the response probability of the delay discrimination session as the prediction variable (Model 2). This model is described as follows:

2 $$\begin{array}{c}{P}_{i}\left(x\right)=\Phi \left(\alpha +{{\alpha }^{d}}_{i}+\left({\beta }^{d}+{{\beta }^{d}}_{i}\right){{P}^{d}}_{i}\left(x\right)\right).\end{array}$$

Here, ${{P}^{d}}_{i}\left(x\right)$ is the probability of the response in delay detection session, $\alpha$ and ${\beta }^{d}$ are fixed-effect parameters of bias and slope of curves, ${{\alpha }^{d}}_{i}$ and ${{\beta }^{d}}_{i}$ are random-effects parameters for bias and slope of curves. Additionally, we used both the amount of visual delay $x$ and response to the visual delay ${{P}^{d}}_{i}\left(x\right)$ to fit the fatigue rate (Model 3) as follows:

3 $$\begin{array}{c}{P}_{i}\left(x\right)=\Phi \left(\alpha +{{\alpha }_{i}+{\alpha }^{d}}_{i}+\left(\beta +{\beta }_{i}\right)x+\left({\beta }^{d}+{{\beta }^{d}}_{i}\right){{P}^{d}}_{i}\left(x\right)\right),\end{array}$$

which includes the fixed and random effects of both $x$ and ${{P}^{d}}_{i}\left(x\right)$ as inputs. To evaluate the prediction performance of these three models, we compared Akaike Information Criteria (AIC) across the models.

To evaluate the level of muscle fatigue electrophysiologically, we calculated the median frequency of the EMG, which is known to decrease with the development of muscle fatigue^34^. The median frequencies were computed from the EMG of the test periods under comparison stimuli. Average values for each visual delay were compared with ANOVA.

To evaluate movement profiles, we computed several indices from the kinematic data. Regarding the data obtained in each phase of the test period, we calculated average values of movement amplitude (difference between maximal and minimum peaks of the finger flexion angle on each movement cycle), movement velocity, movement cycle (interval between timings when flexing finger passed the center of the two target lines), and average-rectified EMG. Since we were interested in how presenting or removing the lag of visual feedback affected movements, we calculated the change in those indices on each trial as

4 $$\begin{array}{c}\Delta I=\frac{{I}_{comparison} \ \ \ - \ \ {I}_{standard} }{{I}_{baseline}}.\end{array}$$

Here, ${I}_{comparison}$ and ${I}_{standard}$ represent the values of each index obtained when comparison and standard stimuli were presented, respectively. A normalizing factor ${I}_{baseline}$ was calculated for each participant by averaging each index in the non-delayed preparation periods. We analysed $\Delta I$ for each index using repeated-measure ANOVAs after averaging for each visual lag.

For Experiment 2, we computed cross-correlation between actual finger movement and the preceding visual feedback to evaluate performance. We evaluated the data of the test period providing − 33 ms and − 50 ms visual feedback in the fatigue evaluation session. The peak value of the cross-correlation and its relative delay was calculated for each trial and then averaged.

## Results

### Experiment 1

**Figure 3:**
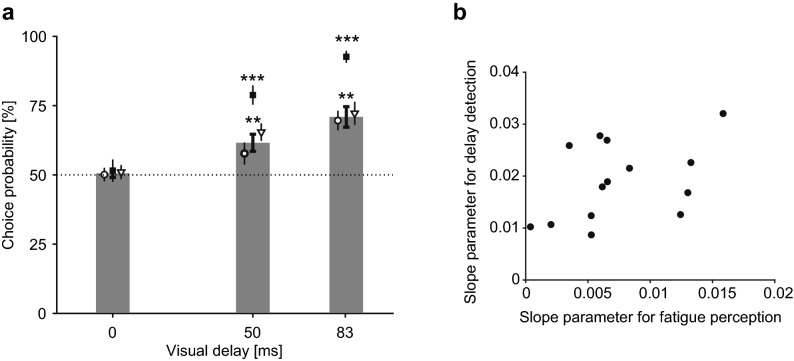
Main results of Experiment 1. (**a**) Choice probability of comparison stimuli. The bar graph shows results of fatigue evaluation session; probabilities of feeling greater muscle fatigue with delayed visual feedback (comparison) than under non-delayed visual feedback (standard). White triangle: sound-on trials; white circle: sound-off trials. Black squares show results of delay detection session; probability of judging comparison stimuli as more delayed than standard. Note that each data point includes responses to stimuli presented during both the first and second phases of test period, thus eliminating the effects of any potential bias due to presentation order within the trial. Each error bar denotes standard error across participants. Asterisks indicate significant difference from chance level after Bonferroni–Holm adjustment. **p < 0.01, ***p < 0.001. (**b**) Relationship between sensitivities of fatigue (sound-off trials) and delay perception across participants. The sensitivities were calculated by fitting the responses against visual delay using probit regression. Each data point represents a single participant. Significant correlation was not found (*r* = 0.42, *p* = 0.13).

**Figure 4:**
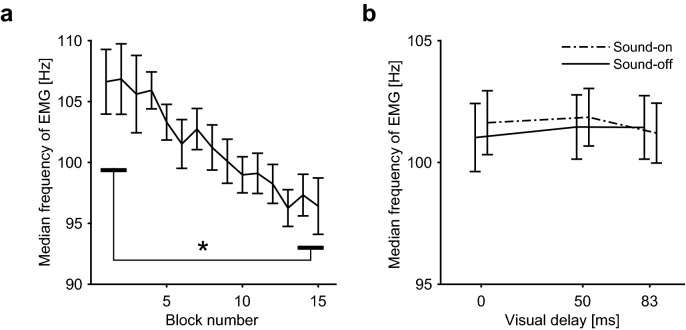
Median frequency of the extensor EMG. (**a**) Mean value for each experimental block. The values were calculated from EMG data during the preparation period. The average value for the last two blocks was significantly lower than that seen for the first two blocks (*t*_(11)_ = 2.77, *p* = 0.018, *d* = 0.80, paired *t*-test). (**b**) Averages for each visual delay condition. Values were calculated from EMG of the test period that showed comparison stimuli. Solid line: sound-off trials; dashed line: sound-on trials. Error bar represents standard error across participants. Two-way ANOVA did not find significant effect of visual delay (*F*_(2,24)_ = 1.28, *p* = 0.30) or sound (*F*_(1,12)_ = 0.99, *p* = 0.34).

We found the choice probabilities were significantly higher than chance (*p* < 0.01) for both 50 ms and 83 ms delays (Fig. 3a), suggesting that the participants felt greater perception of fatigue under the delayed visual feedback. Since the presentation order of the comparison stimuli was pseudo-randomized in each experimental block, the increase in the perception of fatigue should be independent of the temporal trend in actual muscle fatigue and resultant performance fatigability. To verify this assumption, we evaluated the median frequency of EMG, a widely used index of muscle fatigue^34^. The median frequency of finger extensor EMG shifted downward over time (Fig. 4a), and a significant difference was found between the first and last two experimental blocks (*t*_(11)_ = 2.77, *p* = 0.018, *d* = 0.80), suggesting muscle fatigue gradually progressed during the experiment. However, this index did not significantly vary among different visual delay conditions (Fig. 4b , *p* = 0.30). Thus, the observed increase in the perceived fatigue was not ascribed to an actual change in muscle fatigue nor performance fatigability.

One could argue that instead of evaluating muscle fatigue, participants made their choices according to the perceived asynchrony of the visual feedback. To test this possibility, we also measured the ability to detect the delay of visual feedback in a later session. If participants were simply responding to delay in the fatigue task, then their performance should have been similar across the two tasks. However, we found that choice probabilities on delayed visual feedback task were quite different from those for fatigue perception (Fig. 3a). Indeed, post-hoc test showed that choice probabilities for visual delay were significantly higher than those for fatigue perception for 50 ms (*t*_(13)_ = 3.81, *p* = 0.0022, *d* = 1.02) and 83 ms (*t*_(13)_ = 7.97, *p* = 2.3 × 10^–6^,* d* = 2.13). In addition, using the probit regression and GLMM framework (see “Methods” section), we fit the responses during the fatigue evaluation and the delay discrimination task, to compute their sensitivities against visual delay. We found that the standard error of the estimated slope parameter $\beta$ was comparable between fatigue and delay perception, suggesting that the response patterns of fatigue and delay perception were fitted by a psychometric curve with similar estimation accuracy (Table S1 in Supplementary Tables). An analysis across participants revealed that the individual slope parameters of the psychometric curve for fatigue perception ($\beta +{\beta }_{i}$) did not correlate with the slope parameters for the delay detection task (Fig. 3b , *r* = 0.42, *p* = 0.13). This means that participants who detected visual delay well did not necessarily express more fatigue. These results indicate that participants did not make choices merely according to the detected mismatch between their movement and visual feedback.

Moreover, we attempted to fit the data using two additional regression models to examine whether the increase in the fatigue rate could be ascribed to the perception of the visual delay. In contrast to the original hypothesis that the intensity of perceived fatigue increases depending on the amount of visual delay (Model 1), an alternative model assumes that the observed change in fatigue rating depends on the discrimination probability of the delayed visual feedback (Model 2). In addition, we also tested the third model which assumes the fatigue rate is affected by both the amount of visual delay and the discrimination probability of the delayed visual feedback (Model 3). Comparing prediction performance of three models revealed that Model 1 (AIC = 206.4) explains the data better than other models (Model2, AIC = 217.4; Model 3, AIC = 214.4; for more details, see Table S2 in Supplementary Tables), suggesting that the response pattern of the fatigue evaluation is better explained directly by the amount of visual delay, rather than by the discrimination rate of the visual delay. These results are therefore consistent with hypothesis that the enhancement of perceived fatigue is independent of perceiving the visual feedback delay. We assume that both fatigue and delay perception are dependent on the processing of sensory prediction error. The results here emphasize that the modulation of fatigue perception is not a consequence of perceived delay on awareness, but directly caused by the prediction error, which would be subconsciously processed.

**Figure 5:**
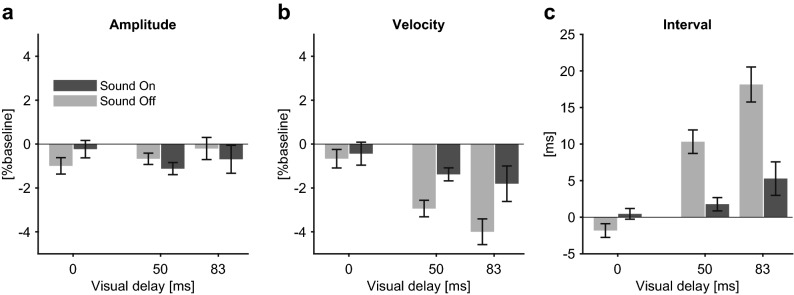
Evaluation of movement profiles. Changes in behavioural indices during test periods ($\Delta I$) in sound-on trials (dark grey) and sound-off trials (light grey). Each value was calculated by subtracting the corresponding index obtained at phases providing the standard stimuli from that for the comparison stimuli, and normalized by the value at baseline (non-delayed preparation period). Error bars are the standard error across participants. (**a**) Movement amplitude. Two-way ANOVA (delay × sound) did not find significant effect of delay (*F*_(2,26)_ = 0.48, *p* = 0.62) nor sound (*F*_(1,13)_ = 0.07, *p* = 0.79). (**b**) Average velocity. Two-way ANOVA found significant effect of delay (*F*_(2,26)_ = 9.00, *p* = 0.0011, partial *η*^2^ = 0.41) and sound (*F*_(1,13)_ = 17.76, *p* = 0.0011, partial *η*^2^ = 0.58). Interaction between delay and sound was not significant (*F*_(2,26)_ = 3.05, *p* = 0.065). (**c**) Movement interval (cycle duration). Two-way ANOVA found significant effect of delay (*F*_(2,26)_ = 22.98, *p* = 1.8 × 10^–6^, partial *η*^2^ = 0.64), sound (*F*_(1,13)_ = 44.22, *p* = 1.6 × 10^–5^, partial *η*^2^ = 0.77), and interaction between delay and sound (*F*_(2,26)_ = 27.97, *p* = 3.3 × 10^–7^, partial *η*^2^ = 0.68).

Another possibility is that participants’ responses reflected changes in motor intensity. If, for example, the amplitude of actual finger movement increased due to the delayed visual feedback, it could bias responses towards feeling more fatigue, because larger movements likely require greater motor effort. To examine this possibility, we compared the amplitude of finger movements between visual delay conditions (Fig. 5). An ANOVA with the factor of visual delay did not find a statistical difference in the movement amplitude (*F*_(2,26)_ = 0.48, *p* = 0.62). As another index of motor intensity, we tested if average-rectified EMG differed with visual delay. Again, ANOVA did not show a significant effect of visual delay on the average-rectified values for both flexor (*F*_(2,26)_ = 0.45, *p* = 0.51) and extensor EMG (*F*_(2,26)_ = 0.34, *p* = 0.71). These results indicate motor intensity did not differ across visual delay, meaning we cannot ascribe the increase in perceived fatigue to changes in motor intensity.

We also checked finger movement speed, finding a decrease in movement velocity (significant effect of visual delay, *F*_(2,26)_ = 9.00, *p* = 0.001, partial *η*^2^ = 0.41) and an increase in movement interval (significant effect of visual delay, *F*_(2,26)_ = 22.98, *p* = 1.8 × 10^–6^, partial *η*^2^ = 0.64) as the visual delay increased, indicating the visual delay slowed finger movements. Slowing of movement, however, was unlikely to explain the increase in the choice rate of perceived fatigue in the delayed conditions. This is because we found that, both for the delay-related decrease in the velocity, and for the delay-related increase in the interval, the results were clearly moderated in sound-on compared to sound-off trials (significant effect of the presence of sound on the velocity, *F*_(1,13)_ = 17.76, *p* = 0.001, partial *η*^2^ = 0.58; significant interaction between delay and presence of sound on the interval, *F*_(2,26)_ = 27.97, *p* = 3.3 × 10^–7^, partial *η*^2^ = 0.68). By contrast, the increase in the rate of perceived fatigue under visual delay did not differ between the sound-on and the sound-off trials (effect of sound, *F*_(1,13)_ = 2.73, *p* = 0.12; interaction between delay and sound, *F*_(2,26)_ = 1.27, *p* = 0.30). Furthermore, to confirm more directly if these variables explain the change in fatigue perception, we performed the fitting of fatigue perception using GLMM with velocity or movement interval as an additional regressor. We found that neither the fixed coefficient of velocity (*t*_(81)_ = 1.08, *p* = 0.28), nor the movement interval (*t*_(81)_ = 1.09, *p* = 0.28) were statistically different from 0, indicating that neither was a significant regressor of fatigue perception. Accordingly, the increase in fatigue rate was unlikely to be caused by the change in the finger movement speed.

### Experiment 2

**Figure 6:**
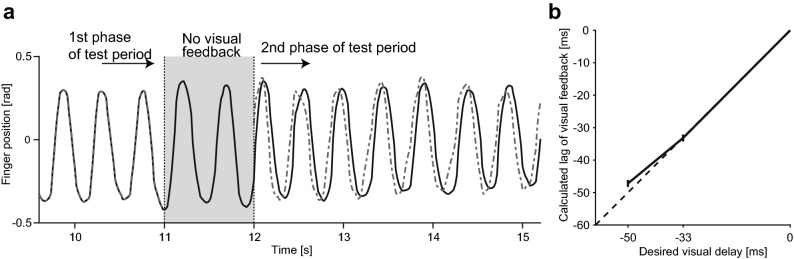
Performance of preceding visual feedback. (**a**) Typical finger trajectory (solid line) and the preceding visual feedback (dashed line) in a single trial of Experiment 2. In the second phase of the test period (t > 12 s), 50 ms of preceding visual feedback was presented. (**b**) Lag of the preceding visual feedback relative to the actual finger movement, estimated by maximizing the cross correlation between actual finger movements and the preceding feedback provided in Experiment 2. Error bars represent standard error across participants.

Experiment 1 suggested that the error between sensory prediction and actual feedback increased perceived fatigue. It is, however, still unclear whether the perceived fatigue always increases with the amount of sensory prediction error or can be reduced if the direction of the prediction error is reversed. To address this point, in the second experiment, we also provided preceding (or negatively lagged) visual feedback as a contrasting manipulation to the delayed visual feedback (Fig. 2). Although it is impossible to generate “negative lag” in the strict sense, we displayed a visual cursor that preceded ongoing finger movement using a prediction technique for human cyclic movements (see Supplementary Information). As shown in Fig. 6a, the preceding visual feedback successfully predicted and preceded participants’ finger movements. Indeed, we obtained high maximal cross-correlation values (0.98 ± 0.00067 for − 33 ms delay trials, and 0.96 ± 0.014 for − 50 ms delay trials) at proper lags (Fig. 6b, − 33.2 ± 1.1 ms for − 33 ms delay trials, and − 47.2 ± 1.1 ms for − 50 ms delay trials) between the preceding visual feedback and actual finger movement trajectory. These results confirm that the preceding visual feedback was capable of predicting participants’ movements with reasonable accuracy and displaying them at the correct timing for our tested conditions.

**Figure 7:**
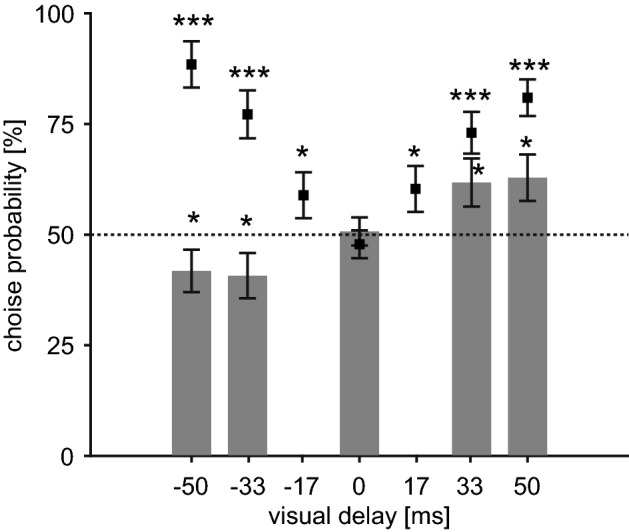
Choice probability of comparison stimuli in Experiment 2. Gray bars show results of fatigue evaluation session; probabilities of response that participants felt more muscle fatigue under comparison stimuli than with standard stimuli. Negative values of the visual delay mean that the preceding visual feedback was provided. Black squares show results of delay detection session; probability of perceiving larger asynchrony of visual feedback with comparison stimuli than standard. Error bars were standard error across participants. Asterisks indicate significant differences from chance after Bonferroni–Holm adjustment. *p < 0.05, ***p < 0.001.

We examined the effect of the preceding visual feedback on fatigue perception by a method similar to Experiment 1 (Fig. 2). The result showed a significant decrease in perceived fatigue with preceding visual feedback (Fig. 7, lower choice probability than chance at − 50 ms and − 83 ms, *p* < 0.05) while delay in visual feedback increased perceived fatigue (higher choice probability than chance at + 50 ms and + 83 ms, *p* < 0.05) as in Experiment 1. This suggests that negative lag of visual feedback causes attenuation rather than enhancement of perceived fatigue. Note that we confirmed that the median frequency of the extensor EMG did not differ across the conditions of visual feedback (effect of visual feedback, *F*_(4,11)_ = 0.59, *p* = 0.67), which implies that the actual condition of muscle fatigue was comparable among the conditions.

We further tested if our manipulations of visual feedback caused detectable sensory prediction error for the participants. We asked participants to evaluate whether the movement of the finger avatar was synchronous with their finger movements. As shown in Fig. 7, the results indicated that the participants detected asynchrony in both preceding and delayed visual feedback (significant difference in choice probabilities from chance) compared to the standard stimuli (non-delayed visual feedback). This suggests that the preceding, as well as the delay of feedback, provides prediction error during the finger movement. Importantly, although both types of visual feedback provided a comparable degree of sensory mismatch for participants, their effect on fatigue perception was opposite between preceding and delay of the feedback.

As in Experiment 1, we fitted the response pattern of fatigue and delay perception to compare their sensitivities against visual delay, including negative lag. The results show that the estimated slope parameters were of a similar value to those obtained from the data in Experiment 1 (Table S1 in Supplementary Tables), suggesting our analyses calculated consistent values across experiments. Again, standard error of the slope parameters was comparable between fatigue and delay perceptions, which further supports that fatigue perception can be fitted using a psychometric function. Furthermore, we found that slope parameters of individual participants were not significantly correlated between fatigue and delay perceptions (*r* = 0.16, *p* = 0.58), thus, we again conclude that perception of fatigue did not directly emerge from the influence of perceived delay on awareness.

## Discussion

Here we examined whether temporal shifts of online visual feedback affect the perception of muscle fatigue during repetitive finger movements. The results showed that perceived fatigue increased under delayed visual feedback. We confirmed that this change in fatigue rating was independent of the physiological condition of muscle fatigue or the level of motor intensity, thus indicating that the modulation of fatigue occurred at the perceptual level. We also found that perceived fatigue decreased with preceding visual feedback, which artificially provided a negative lag between ongoing movement and visual feedback. Accordingly, our study demonstrated that perception of muscle fatigue is modulated by temporal errors inserted into feedback of self-movements. These results suggest that the brain ascribes prediction errors to the fatigue of one’s body, as it does for other extrinsic factors.

Previous studies have suggested that prediction errors play a critical role in discriminating externally-produced stimuli from the sensory consequences of one’s own action^7,8^. In addition, it has been proposed that through an inverse estimation from the information contained in the prediction error, the brain computes the properties of the external factors causing the error^15,16,37^. Whereas these studies have demonstrated the function of prediction errors when processing external causes, recent studies regarding interoceptive inference^38^ have suggested that errors between predicted internal state of the body and interoceptive signals may contribute to estimating and regulating the bodily condition^19,20^. However, it remains unexplored whether the brain also attributes the prediction error calculated from exteroceptive or proprioceptive signals to changes in intrinsic factors. We reasoned that if an appropriate context was given, these action-related prediction errors will be used to monitor changes in the intrinsic condition that affect motor function, such as injury or fatigue. Indeed, our experiment showed that temporal delay in visual feedback increased perceived fatigue. This result suggests that the brain may have related the prediction error to performance fatigability due to the progress of muscle fatigue and then modulated the intensity of perceived fatigue to reflect the estimated change in motor function.

In the motor control domain, ascribing prediction error to an appropriate cause is a critical problem to solve for efficient motor learning. This is because the manner in which internal models should be maintained and updated largely depends on identifying the factor that caused a given prediction error^39–41^. For example, the proper learning strategy would be different in cases where the error is caused by a change in body dynamics, as opposed to cases where it is caused by misestimating the properties of hand-held tools, or merely by noise in the sensorimotor system. It has been proposed that the brain estimates the probable cause of the prediction error by considering the context and statistical characteristics of the error to determine how to update the internal model^40,42^. Possibly, also regarding the perceptual system, the brain determines which factor caused a prediction error through a similar computational process, rather than exclusively attributing the error to external causes, thereby suitably modulating perception for the situation. In this study, we increased the level of background fatigue and induced clear performance fatigability by intensive movement before the tasks and kept the participant’s finger from touching any objects. Through these experimental controls, we attempted to provide the participants with a context in which muscle fatigue, rather than other factors, had a high probability of affecting motor performance. It is likely that, affected by these contextual cues, the brain associates prediction error with one’s own fatigability condition.

In some cases, it is possible that the brain cannot readily find an appropriate association between prediction error and its cause. For instance, involuntary or automatic actions are executed without the cortical process for computing motor commands. It has been suggested that this class of movements is not accompanied by an efference copy, thus, prediction errors emerge^43–45^ even if neither extrinsic nor intrinsic factors disturb body movement. Interestingly, such automatic movements sometimes yield hard-to-explain, odd sensations^46,47^. Potentially, in accordance with our hypothesis, this phenomenon might be accounted for by a failure to attribute the prediction error. Specifically, the brain may fail to assign the observed error to the proper perceptual attributes, due to the difficulty of finding any internal or external causes suitable to the context.

Our findings also offer insights into the mechanism of perceiving muscle fatigue, reflecting the current motor condition. Muscle fatigue is characterized by a reduction of muscular output due to sustained motor activity, namely performance fatigability. It has been shown that muscle fatigue is caused by complex mechanisms^26,27,48,49^, including metabolic changes in muscle fibres, reduction in the efficacy of neuromuscular transmission, and other supraspinal factors, which eventually cause a motor error and deteriorate task performance. Though several studies have shown cortical areas responsible for subjective feelings about exercise-induced fatigue^50–52^, computational accounts of how the perception of fatigue emerges are still insufficient. Our results showed that the perceived intensity of muscle fatigue was modulated by prediction errors relating to feedback from limb movement, although the actual fatigability condition was supposed to be unchanged. Thus, this finding suggests that the brain estimates muscle fatigue by detecting reduction of motor output using sensory prediction error, in addition to monitoring the physiological condition of the body through afferent signals from muscles and other interoceptive information. Previous studies have suggested that ‘perceived effort’ reflecting the magnitude of the central motor drive is involved in the perception process of muscle fatigue^53^. In contrast, several recent studies have proposed that errors between expected and actual action contribute to the generation of fatigue perception^52,54^ in addition to interoceptive signals^55^ and their prediction errors^21^. In agreement with these latter ideas, our results demonstrate the contribution of sensory prediction error to the perception of muscle fatigue. We showed modulation of perceived fatigue merely by inducing error in visual feedback without changing motor intensity.

In many past experiments, temporal delay has been used as a method of inducing a mismatch between action and its sensory feedback. Delay in online visual feedback of one’s movement not only affects the perceived physical properties of external objects, but also disrupts motor control because it provides error in the monitoring of current body state^56^. Indeed, we found a small but significant effect of delayed visual feedback on movement (decrease in movement speed and extension of movement cycle) as previously reported^57,58^. Given that the decrease in movement speed was observed even in contexts without muscle fatigue, the motor slowing possibly occurred due to the interaction between delayed visual feedback and the motor control system, rather than a change in fatigability. Furthermore, our analyses showed that this change in movement did not explain modulations of perceived fatigue, rendering the claim that perceptual modulation occurs due to changes in motor behaviours unlikely.

Meanwhile, partly due to its technical difficulty, it is relatively unclarified how our perceptual system processes situations where sensory feedback precedes voluntary actions. An earlier study showed that participants tended to perceive sensory stimuli provided in advance of finger tapping timing as generated by an external source^10^, as in a delayed feedback condition. Conversely, we provided online visual feedback that proceeded self-movement via a unique method exploiting a signal prediction technique. This showed that preceding visual feedback affects the intensity of perceived fatigue, suggesting that, in our experimental setup, the brain also ascribed prediction error to muscle fatigue even if the direction of the error was opposite to the delayed feedback (i.e., negative lag).

Importantly, the intensity of perceived fatigue was attenuated with preceding visual feedback, whereas it was enhanced with delayed visual feedback. These results show that the modulation of fatigue perception is affected not only by the extent of prediction error, but also by its temporal direction. Possibly, when sensory feedback of body movement runs behind the prediction, the brain attributes prediction error to a reduction in motor output, resulting in upward correction of perceived fatigue. Conversely, if sensory input indicates that body movement precedes the prediction, the prediction error would be ascribed to an overestimation of fatigue level, resulting in downward correction of perceived fatigue. Considering that human observers have the ability to compute force information from visual body motion^16^, the brain would estimate muscle fatigue by associating the spatiotemporal pattern of prediction errors to a change in muscle condition, rather than by simply detecting temporal gaps between sensory prediction and actual feedback. Future studies should clarify what aspect of the spatiotemporal pattern of a prediction error (e.g., the error of position, velocity, or acceleration) contribute to fatigue perception, so a detailed computational account can be developed.

As a limitation of the present study, the preceding visual feedback did not virtually precede actual body movement in a strict sense. Since the experimental setup included the unavoidable system delay of 57 ms (see “Methods” section), even in both the − 30 ms and − 50 ms negative lag conditions, the provided visual feedback was in fact physically delayed from actual finger movement. Given this, the interpretation of the results may not be straightforward. It is known that exposure to delayed sensory feedback causes an adaptation of the temporal synchrony between action and sensory feedback^59^. In the present study, since participants had sufficient experience of the visually guided finger movement under the system delay for such adaptation to take place, it is supposed that for the 0 ms delay condition, visual feedback was indeed perceived as synchronized with self-action, despite it being physically delayed (i.e., the system delay). This interpretation is consistent with the result that participants perceived the 0 ms delay condition as the most synchronous, as shown in Fig. 7. In fact, a previous study suggested that internal sensory prediction is affected by sensorimotor recalibration and that prediction error is calculated based on the adapted synchrony between action and feedback, rather than a physical temporal mismatch^9^. Accordingly, we think that the negative lag condition in the present study introduced the sign-inverted prediction error as we expected, which caused the decrease in perceived fatigue.

In conclusion, the results of present study suggest that information contained in prediction errors is used to estimate the condition of the body, accounting for intrinsic factors such as muscle fatigue. This finding supports the idea that, dependent on context, the brain flexibly attributes prediction errors, thereby properly correcting the perception of disturbances of the sensorimotor state, regardless of whether they are caused by extrinsic or intrinsic factors.

## Supplementary Information

Supplementary Information.

Supplementary Tables.

## Data Availability

The datasets generated and/or analyzed during the current study are available from the corresponding author on reasonable request.
