# Supplementary material for: Attribution of sensory prediction error to perception of muscle fatigue
Source: Sci Rep. 2022 Oct 6;12:16708. doi: 10.1038/s41598-022-20765-9 (PMC9537327; doi:10.1038/s41598-022-20765-9)
Supplement: Supplementary file 1 — Supplementary Information. [file 41598_2022_20765_MOESM1_ESM.pdf]

Supplementary methods

Here we will describe the method for generating the online preceding visual feedback (negative lag) of the cyclic finger movement. We employed a real-time prediction technique for human cyclic movements[1] with a slight modification.

As training data of a predictor, we recorded m $(> n)$ samples of a finger movement (joint angle of the index finger) $\mathbf{y} = [y_1 \cdots y_{\mathrm{m}}]^T$, where T_s is sampling time and nT_s is cycle duration of the finger movement. Considering periodicity of the training data $\mathbf{y}$, we define cyclic state variables $\mathbf{x}_i$ as follows:

$$\mathbf{X} = [\mathbf{x}_1 \cdots \mathbf{x}_m],$$

$$\mathbf{x}_i = [\cos(i\omega_0) \quad \sin(i\omega_0)]^T (i = 1, 2, \cdots m), \tag{1}$$

where $\omega_0 = 2\pi/n$. It is assumed that data samples y_i were generated from $\mathbf{x}_i$ as an equation $y_i = f(\mathbf{x}_i) + \epsilon$, where ϵ is zero-mean Gaussian noise with variance $\sigma_n{}^2$. By fitting this relationship between $\mathbf{y}$ and $\mathbf{X}$ with Gaussian process regression[2], we obtain a predictive distribution over its output y_* to an arbitrary input $\mathbf{x}_*$ as a Gaussian distribution

$$p(y_*|\mathbf{x}_*) = N(\mu_{y_*}, \Sigma_{y_*}) \tag{2}$$

where mean and variance are as follows:

$$\mu_{y_*}(\mathbf{x}_*|\mathbf{X}, \mathbf{y}) = \mathbf{k}_*^T[\mathbf{K}(\mathbf{X}, \mathbf{X}) - \sigma_n \mathbf{I}]^{-1}\mathbf{y}, \tag{3}$$

$$\Sigma_{y_*}(\mathbf{x}_*|\mathbf{X}, \mathbf{y}) = k(\mathbf{x}_*, \mathbf{x}_*) - \mathbf{k}_*^T[\mathbf{K}(\mathbf{X}, \mathbf{X}) - \sigma_n \mathbf{I}]^{-1}\mathbf{k}_*. \tag{4}$$

Here, Gaussian kernel k and the vector $\mathbf{k}_*$ are defined as $k(\mathbf{x}, \mathbf{x}') = \sigma_f \exp\left(-\frac{1}{2l^2}\|\mathbf{x} - \mathbf{x}'\|^2\right)$ and $\mathbf{k}_* = [k(\mathbf{x}_*, \mathbf{x}_1) \cdots k(\mathbf{x}_*, \mathbf{x}_n)]^T$, respectively. Gramian matrix $\mathbf{K}(\mathbf{X}, \mathbf{X})$ is defined as $K[p, q] = k(\mathbf{x}_p, \mathbf{x}_q)$. Note that σ_f, l, and σ_n are hyperparameters. We expect that the distribution of cyclic finger movement following

the recorded training data should similarly be described by equations (3) and (4) if we assume that the shape of the movement pattern does not drastically change during each experimental block. However, since it is obvious that repetition of movement causes both spatial and temporal variation in the movement pattern, we define additional state variables describing those variations. Here, state vectors at time t are set as follows:

$$\boldsymbol{\theta}_t = [\phi_t \ \omega_t \ a_t \ b_t]^T \tag{5}$$

$$\mathbf{x}_t = [\cos(\phi_t) \quad \sin(\phi_t)]^T. \tag{6}$$

In equation (5), a state variable ϕ_t is a phase, which holds the periodicity of the pattern through equation (6), and ω_t represents the transition speed of the phase. Note that ω_t does not affect the output directly. a_t and b_t are amplitude and bias, which denote the time-depending variation of spatial scaling and shift of the movement pattern. As equation (4) and (5), we assume that observed finger position z_t given the state vector $\boldsymbol{\theta}_t$ is distributed normally as

$$p(z_t|\boldsymbol{\theta}_t) = N\left(\mu_{z_t}, \Sigma_{z_t}\right), \tag{7}$$

where, mean and variance are written with a_t and b_t as follows:

$$\mu_{z_t} = a_t \cdot \mu_{y_*}(\mathbf{x}_*|\mathbf{X}, \mathbf{y})\big|_{\mathbf{x}_*=\mathbf{x}_t} + b_t \tag{8}$$

$$\Sigma_{z_t} = {a_t}^2 \cdot \Sigma_{y_*}(\mathbf{x}_*|\mathbf{X}, \mathbf{y})\big|_{\mathbf{x}_*=\mathbf{x}_t}. \tag{9}$$

Note that equation (7) is obtained as a linear transformation of equation (2). Although we cannot directly observe the state variables in equation (5), introducing Kalman filter enables us to estimate and predict those state variables from observed data sample z_t at each time step. Here, we assumed a model for transition of the state vector $\boldsymbol{\theta}_t$ over discrete time as follows:

$$\boldsymbol{\theta}_t = \mathbf{F}\boldsymbol{\theta}_{t-1} + \mathbf{w}_t \tag{10}$$

$$z_t = \mathbf{H}_t \boldsymbol{\theta}_t + \mathbf{v}_t, \tag{11}$$

where transition matrix $\mathbf{F}$ is defined as

$$\mathbf{F} = \begin{bmatrix} 1 & 1 & 0 & 0 \\ 0 & 1 & 0 & 0 \\ 0 & 0 & 1 & 0 \\ 0 & 0 & 0 & 1 \end{bmatrix}.$$

In equation (10), process noise $\mathbf{w}_t$ is Gaussian noise distributed with $N(0, \mathbf{Q})$. Note that, since the covariance matrix $\mathbf{Q}$ works as a weight matrix, we can tune the performance of the Kalman filter by changing its value. Observation noise $\mathbf{v}_t$ is also Gaussian noise whose mean and covariance are $\mathbf{0}$ and $\mathbf{R}_t = \Sigma_{z_t}$. Since the mapping from the state vector $\boldsymbol{\theta}_t$ to an output z_t in equation (8) is nonlinear, we compute Jacobian matrix and use it in the Kalman filter instead of equation (8)[3]. By linearizing the equation (8), we get $\mathbf{H}_t$ as

$$\mathbf{H}_t = \frac{\partial \mu_{z_t}}{\partial \boldsymbol{\theta}_t} = \begin{bmatrix} \frac{\partial \mu_{z_t}}{\partial \mathbf{x}_t} \cdot \frac{d\mathbf{x}_t}{d\phi_t} & 0 & \mu_{y_*}(\mathbf{x}_* | \mathbf{X}, \mathbf{y})\big|_{\mathbf{x}_* = \mathbf{x}_t} & 1 \end{bmatrix}. \tag{12}$$

Using the Kalman filter with equation (10) and (11) with appropriate initial values, we can repeat state estimation and one-step prediction from an obtained sample z_t at each time. In addition, it is also possible to make a multi-step prediction with equation (10). Predicted movement data at time $t + s$ based on information at time t is obtained as

$$\hat{z}_{t+s|t} = a_t \cdot \mu_{y_*}(\mathbf{x}_* | \mathbf{X}, \mathbf{y})\big|_{\mathbf{x}_* = \hat{\mathbf{x}}_{t+s|t}} + b_t, \tag{13}$$

where,

$$\hat{\mathbf{x}}_{t+s|t} = [\cos(\phi_t + s\omega_0) \quad \sin(\phi_t + s\omega_0)]^T.$$

Thus, the online preceding visual feedback is achieved with the predicted finger position $\hat{z}_{t+s|t}$ in equation (13).

In the actual experiment, we recorded three cycles of finger movement (thus, $m = 3n$) synchronous to beep sound just before each experimental block started. We

used it to drive the predictor through the subsequent block, where $T_s = 0.0167$ [s], $n = 28,\ m = 3n$. We also used the following parameters: initial value of state vector, $\boldsymbol{\theta_0} = [0 \quad \omega_0 \quad 1 \quad 0]^T$; covariance of process noise, $\mathbf{Q} = diag([2 \times 10^{-4}\ \ 10^{-1}\ \ 2 \times 10^{-1}\ \ 10^{-2}])$; and hyperparameters, $(\sigma_f,\ l,\ \sigma_n) = (2,\ 0.5,\ 0.5)$.

We found that once the state variable ω_t is estimated to a quite small value, the predictor no longer can properly estimate the state vector. Thus, if ω_t became less than a threshold value (ω_t < 0.055), we regraded the trial as failed, and discarded it from subsequent analyses.

Supplementary References

1. Matsubara, T., Hyon, S.-H. & Morimoto, J. On-line Stylistic Predictionfor Human Periodic Motions. *Neurosci. Res.* **68**, e217–e218 (2010).
2. Rasmussen, C. E. & Williams, C. K. I. *Gaussian Processes for Machine Learning*. (MIT Press, 2006).
3. Ko, J. & Fox, D. GP-BayesFilters: Bayesian filtering using Gaussian process prediction and observation models. *2008 IEEE/RSJ Int. Conf. Intell. Robot. Syst.* **27**, 3471–3476 (2008).
