# Supplementary material for: Attribution of sensory prediction error to perception of muscle fatigue
Source: Sci Rep. 2022 Oct 6;12:16708. doi: 10.1038/s41598-022-20765-9 (PMC9537327; doi:10.1038/s41598-022-20765-9)
Supplement: Supplementary file 2 — Supplementary Tables. [file 41598_2022_20765_MOESM2_ESM.pdf]

Supplementary tables

Table S1. Fitting parameters of fatigue and delay perception against visual delay.

Model	df	α	Standard error of α	P($\alpha \neq 0$)	β	Standard error of β	P($\beta \neq 0$)	SD of β_i
**(Exp. 1)**								
**Fatigue**	40	0.030	0.078	0.71	0.0075	0.0019	0.00028	0.0050
**Delay**	40	-0.018	0.093	0.093	0.019	0.0029	5.0×10^{-8}	0.0084
**(Exp. 2)**								
**Fatigue**	68	0.050	0.048	0.31	0.0064	0.0016	0.00014	0.0052
**Delay**	96	0.050	0.059	0.41	0.020	0.0026	9.3×10^{-9}	0.0084

Table S2. Comparison of model parameters for fitting in Experiment 1.

	df	α	P($\alpha \neq 0$)	β	P($\beta \neq 0$)	β^d	P($\beta^d \neq 0$)	AIC
**Model 1**	40	0.030	0.71	0.0075	0.00028	—	—	206.4
**Model 2**	40	-0.37	0.036	—	—	1.03	0.00024	217.4
**Model 3**	39	0.02	0.91	0.0074	0.014	0.0096	0.98	214.4
